# Preparation of morphology-controllable PGMA-DVB microspheres by introducing Span 80 into seed emulsion polymerization

**DOI:** 10.1039/c7ra13158e

**Published:** 2018-01-10

**Authors:** Hailin Cong, Bing Yu, Lilong Gao, Bo Yang, Fei Gao, Hongbo Zhang, Yangchun Liu

**Affiliations:** Institute of Biomedical Materials and Engineering, College of Chemistry and Chemical Engineering, Qingdao University Qingdao 266071 China yubingqdu@yahoo.com +86-532-85955529 +86-532-85953995; Laboratory for New Fiber Materials and Modern Textile, Growing Base for State Key Laboratory, College of Materials Science and Engineering, Qingdao University Qingdao 266071 China

## Abstract

Microporous, hollow, or macroporous polymer spheres were prepared by a seed emulsion polymerisation method. Different from the conventional seeded emulsion polymerization, the sorbitan monooleate (Span 80) was added to the seeded emulsion polymerization. In this study, the monodisperse PS seeds prepared by dispersion polymerization were swelled by dibutyl phthalate (DBP), glycidyl methacrylate (GMA), divinylbenzene (DVB) and Span 80 successively. The effect of the amount of Span 80 on the morphology of microspheres was investigated. As different amount of Span 80 was added to the mixture, the poly(glycidyl methacrylate-divinylbenzene) (PGMA-DVB) microspheres showed a variety of morphologies containing microporous, hollow, and macroporous structure. In addition, uniform hollow particles with different pore size can be obtained through adjusting the amount of Span 80.

## Abstract 2

The obtained PGMA-DVB microspheres showed a variety of morphologies by adjusting the amount of Span 80 in the seeded emulsion polymerization.

## Graphical abstract

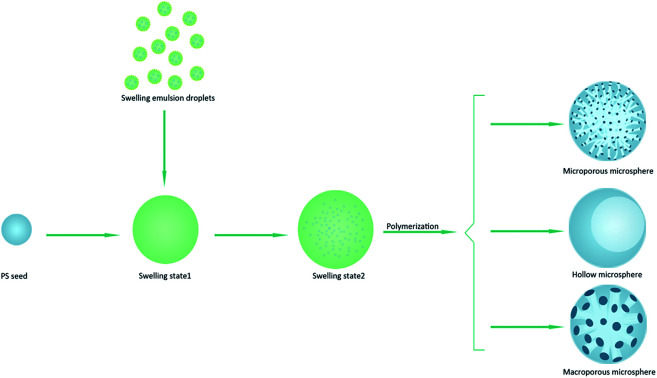

## 1. Introduction

Polymer microspheres are widely applied in many fields due to their special characteristics, such as small size, large specific surface area, high diffusibility and mobility. Porous and hollow polymer microspheres have drawn great attention.^1,2^ Porous microspheres are classified into three types according to their pore size: micro-porous microspheres, meso-porous microspheres and macro-porous microspheres. These porous particles with large surface area have the ability to uptake various solvents with different polarity, and have been widely used in drug delivery, chromatography and molecular imprinting.^3–11^ Hollow particles with a hollow structure and high loading capacity are usually used in controlled drug delivery, cell sorting and catalysis.^12–14^

The polymer microspheres are mainly prepared by heterogeneous polymerizations due to the sphere with the lowest surface to volume ratio in the heterogeneous system. Several methods can be used to prepare porous polymer microsphere, such as dispersion polymerization, emulsion polymerization, precipitation polymerization.^15–17^ The porogen is the critical factor to yield the porous structure in the preparation of porous microsphere. Although the porogens do not react with monomer, crosslinking agent or initiator, they occupy the space and control the morphology and the formation time of microgel particles in the polymerization. For preparing hollow spheres, the template method is an intriguing strategy. The types of templates are various, such as polymer latexes, silica spheres, and even water droplets all can be used as template.^18–20^ The interactions between monomer/polymer chain and the template make them attach each other. After polymerization, the templates are removed and then the hollow polymer microspheres are obtained. This method requires there are the interactions between template and monomer/polymer chain which ensure the shell formed by the polymer to attach to the template. So the kinds of the monomer are limited.

Sorbitan monooleate (Span 80) as oil-soluble surfactant can form inverse micelles with water or water-soluble monomer. And it is usually used in inverse emulsion polymerization and high internal phase emulsion.^21–26^ In recent years, Span 80 as porogen was applied in suspension and polymerization emulsion to prepare porous polymer microsphere.^27–29^ Especially, work of Zhou *et al.* proved that the oil phase could absorb water from the aqueous phase due to the presence of Span 80.^28,29^ Compared with porous polymer microsphere prepared by phase separation in oil phase, the water was introduced in polymerization system and increased the space occupied by the porogen. The obtained microsphere possessed macroporous structure and the pore size reached to 500 nm. But to the best of our knowledge, there is no reported example of utilization of the Span 80 in the seeded emulsion polymerization. In this study, the Span 80 was introduced into the seeded emulsion polymerization to research the effect of Span 80 on the morphology of PGMA-DVB particles. GMA and DVB were selected as monomer and crosslinker to synthesize microspheres due to its good ability to swell seed beads.^30–33^ While different amount of Span 80 was added to the reaction system, hollow, microporous or macroporous structure microspheres could be formed. Additionally, the shape and uniformity of the obtained microspheres were greatly improved compared with previous reports. Potentially, these as-prepared porous PGMA microspheres with large amounts of epoxy group could be further modified by amine–epoxy, thiol–epoxy, azide–epoxy, acid–epoxy, or hydrolysis reactions.^34–40^ For instance, fluorescent molecules with thiol group could be used to graft to the surface of microspheres for fluorescence imagining. What's more, the generated hydroxy group can be reacted in a sequential manner and bi-functional spheres can be accessed.^41–43^

## 2. Experimental

### 2.1 Materials

Styrene (St, Tianjin Chemical Company) was distilled under reduced pressure. Sodium dodecylsulfate (SDS, 99%), poly(*N*-vinylpyrrolidone) (PVP, *M*_n_ = 40 000) and glycidyl methacrylate (GMA) were purchased from Aladdin Chemical Reagent Company. 2,2-Azobis(isobutyronitrile) (AIBN, 98%), dibutyl phthalate (DBP, 99%), tetrahydrofuran (THF, 99%) were purchased from Tianjin Fuyu Fine Chemical Company. Polyvinyl alcohol (PVA, *n* = 1750 ± 50) and Span 80 was provided by Tianjin Guangfu Chemical Company. Benzoyl peroxide (BPO, 95%) was supplied from Tianjin Beichen Chemical Company. Divinylbenzene (DVB, 80%) were obtained from Shanghai Macklin Biochemical Company.

### 2.2 Preparation of polystyrene (PS) seed particles

The PS seeds were prepared by dispersion polymerization method as described previously.^44^ Briefly, 10 mL of St, 0.44 g of AIBN and 1 g of PVP as monomer, initiator and steric stabilizer, respectively, were dissolved in 80 g of ethanol in a three-neck flask equipped with IKA RW20 mechanical stirrer under the nitrogen atmosphere. The polymerization proceeded at 70 °C for 24 h with stirring at 120 rpm. Then the particles were washed by ethanol and dried under a vacuum at room temperature.

### 2.3 Synthesis PGMA-DVB particles

In this experiment, the monodisperse PGMA-DVB particles were prepared by multistage polymerization emulsion method. Firstly, the PS seeds (0.26 g) were dispersed in 10 mL distilled water. The resultant mixture containing 1.2 mL of DBP and 20 mL of 0.375% SDS (w/w) aqueous solution was emulsified by sonication. The sonication was carried out with an ultrasonic homogenizer (BILON92-II, China). Then both seeds dispersion and the resulting emulsion were added into the three-flask equipped with IKA RW20 mechanical stirrer under the nitrogen atmosphere at 30 °C for 24 h with stirring at 300 rpm. Second, the oil phase containing 0.6 mL of GMA, 2 mL of DVB, 0.12 g of BPO and the different amount of Span 80 were added in 30 mL of 0.25% SDS (w/w) aqueous solution. The suspension was also emulsified by the same method as the first step. The obtained emulsion were added into the flask together and swelled under the same condition for 24 h. Then 3.5 mL of 10% PVA (w/w) solution were added into the flask. The polymerization reaction was carried out at 70 °C for 24 h with stirring at 120 rpm. After polymerization, the obtained particles were centrifuged and washed with ethanol for three times. The porogens and unreacted regent were extracted by THF through heating circumfluence at 60 °C for 24 h. Then the PGMA-DVB beads were centrifuged and washed with ethanol again and dried under a vacuum at room temperature.

### 2.4 Characterization

The surface morphology and structure of the PS seed particles and PGMA-DVB spheres were observed by scanning electron microscopy (SEM, JEOL JSM-6309LV) and transmission electron microscopy (TEM, JEOL JEM-1200). An accelerated surface area and porosimetry analyzer (ASAP, Micromeritics 2020) was used for the BET tests.

## 3. Results and discussion

### 3.1 The preparation of PS seeds

**Fig. 1:**
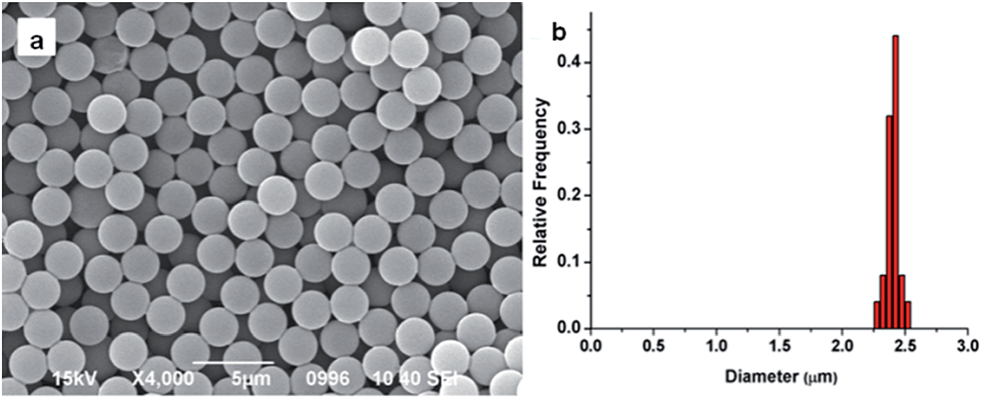
SEM images of PS seed microspheres (a) and the histogram of the distribution of PS seed (b).

Conventional dispersion polymerization method was chosen to prepare the monodisperse PS seeds in this experiment, for its outstanding superiority in particle diameter controlling.^45^ The size and monodispersity of obtained particles were characterized by the SEM. As shown in Fig. 1, the PS seeds were about 2.4 μm and the coefficient of variance was 2.2%. The PGMA-DVB microspheres were obtained by multi-step swelling methods of PS seeds. Using the obtained PS seeds as swelling templates could ensure the obtained microspheres possess good uniformity.^46^

### 3.2 Effect of Span 80 amount on morphology of PGMA-DVB microspheres

The PGMA-DVB particles were prepared by multi-step swelling polymerization which was based on the multistep swelling of PS seeds. First, the PS seeds absorbed hydrophobic agent (DBP) from emulsion and then swelling in a mixture of monomer GMA, crosslinking agent DVB, initiator BPO and Span 80. As described in the previous work,^28,29^ the concentration of Span 80 significantly affected the structure of the microspheres. So the concentration of Span 80 as the primary factor was estimated in this study. We controlled the concentration of Span 80 by adjusting the amount of Span 80 in the seeded emulsion polymerization. The serial amount gradient we chosen were 0 mL, 0.3 mL, 0.6 mL, 0.9 mL, 1.2 mL, 1.5 mL, and 1.8 mL.

**Fig. 2:**
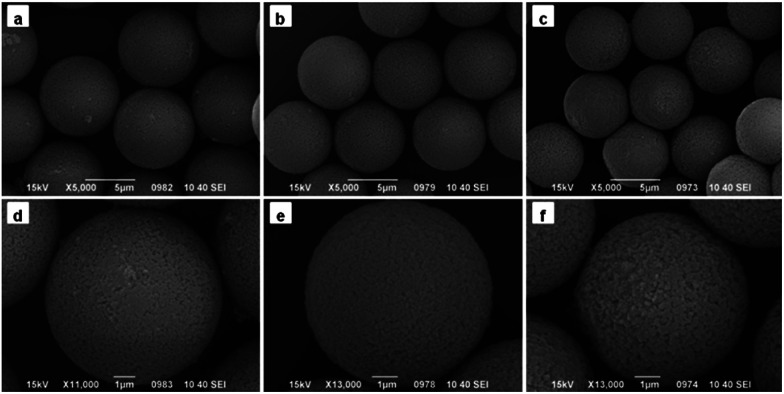
SEM images of PGMA-DVB microspheres with different addition amount of Span 80: (a and d) 0 mL, (b and e) 0.3 mL, and (c and f) 0.6 mL.

As shown in Fig. 2, the obtained PGMA-DVB particles were well-distributed and possessed porous structure when the amount of Span 80 was below 0.6 mL. Nitrogen gas adsorption–desorption measurements with theoretical BET analysis were applied to further explore the differences among the three kinds microspheres. The results were shown in Table 1. It was shown that the pore size distributions and BET surface area depending on the amount of Span 80. The pore size increased as the increase amount of Span 80. But there was trade-off between specific surface area and the amount of Span 80. Obviously, nanoparticles could be filled into the pores of PGMA-DVB through subsequent processes such as modification, adsorption and precipitation. For instance, the introduction of Fe_3_O_4_ nanoparticles, fluorescent molecules, catalyst or proteins may help us to obtain a series of different functional hybrid microspheres.^42,47–49^

**Table 1:** Porosimetry measurement results of the microspheres prepared with different addition amount of Span 80

Amount of Span 80 (mL)	BET surface area (m^2^ g^−1^)	Average pore size (nm)
0	325.1388	10.6727
0.3	291.1630	11.1412
0.6	192.1760	12.0489

**Fig. 3:**
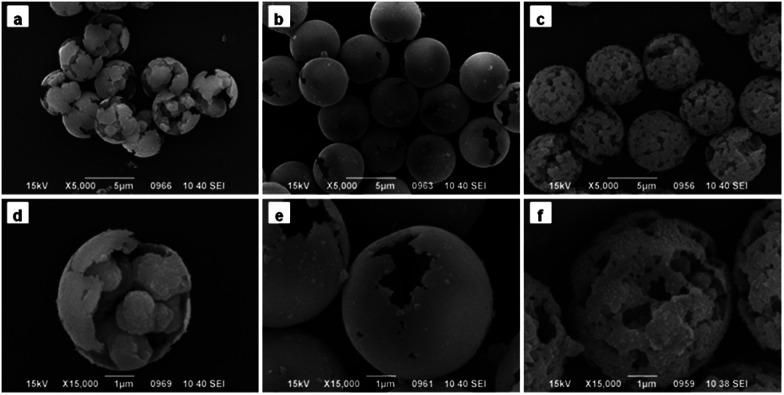
SEM images of PGMA-DVB microspheres with different addition amount of Span 80: (a and d) 0.9 mL, (b and e) 1.2 mL, and (c and f) 1.5 mL.

**Fig. 4:**
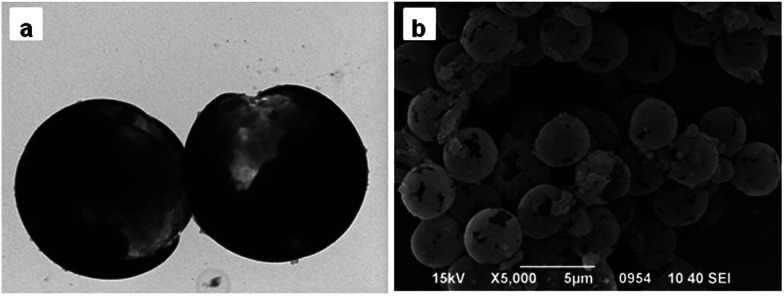
TEM image of PGMA-DVB microspheres with addition of 1.2 mL Span 80 (a) and SEM image of PGMA-DVB microspheres with addition of 1.8 mL Span 80 (b).

When the amount of Span 80 was reached to 0.9 mL, the morphology of particles changed dramatically. As show in Fig. 3a, pore structure disappeared and the shape of obtained microspheres was like broken eggs. While the amount of Span 80 was continuously increased to 1.2 mL, the morphology was completely different from that previously described.^28,29^ As seen in Fig. 3b, there was a gap existing in the microspheres instead of micropores. When viewed in the larger multiples, the internal of particles seemed to be hollow. TEM was utilized to confirm this speculation. Fig. 4a indicated that the interior of the microspheres was hollow and the shell in the neighboring region of breach was much thinner than the average thickness. As the amount of Span 80 reached to 1.5 mL, the microsphere possessed porous structure again, as shown in Fig. 3c. But the pore size of it was much bigger than that of Fig. 2. Then we adjusted the addition amount of Span 80 to 1.8 mL, the image of obtained microspheres was illustrated in Fig. 4b. The obtained particles were much smaller than the above microspheres.

### 3.3 Formation mechanism of microspheres with different morphologies

**Fig. 5:**
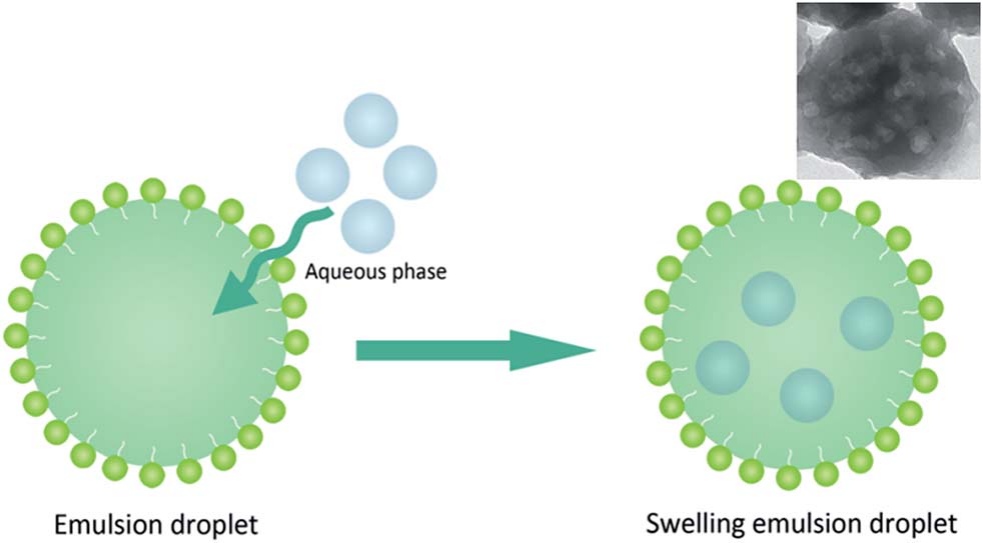
Schematic illustration of forming swollen micelles with H_2_O droplets.

From the above description, the obtained PGMA-DVB microspheres showed microporous, macroporous and hollow morphologies. It was apparent that the morphologies of microsphere were closely related to the concentration of Span 80. The emulsion of Span 80, monomer, crosslinker and initiator was utilized to swell the first swelling particles. In this experiment, the quality of each component was determined except Span 80. Although the work of Zhou *et al.* had proved that the absorption of Span 80 endued the swelling particles the ability to acquire water from the aqueous phase besides occupy space in the polymerization system, there was still a great difference between our work and his work.^28,29^ In Zhou's work, the Span 80 was homogeneously mixed with monomer phase. The water was directly introduced into polymerization system. However in our work, the swelling particles obtained the Span 80 by absorption of monomer phase emulsion droplets. Monomer phase emulsion droplets not only endowed the swelling particles the water absorption capacity, but absorbed water themselves. In others words, it takes several hours to complete the swelling procedure, and some monomer phase emulsion droplets packing water entered into the swelling particles.^50–52^ In swelling procedure, there were some monomer phase emulsion droplets which could absorb water by themselves due to the presence of Span 80 before they were absorbed by swelling PS seeds (as illustrated in Fig. 5). But the droplets were prepared by an ultrasonic homogenizer whose power reached to 600 W. And its large power led the size of the droplets too small to be observed by light-microscope.^53^ To confirm this, the monomer phase emulsion droplets were directly used to polymerize. It could be found from the TEM picture of obtained particles that there were cavities presenting in the particles. This explained that water droplets could be packed into monomer phase emulsion droplets. Then the first swelling absorbed the monomer phase emulsion droplets. And the presence of Span 80 boosted the water and oil phase formed O/W micelles at non-aqueous internal of swelling particles. The structure of second swelling particles with H_2_O droplets was W/O/W which was similar to double emulsion.

#### 3.3.1 Formation of microspheres with hollow structure

**Fig. 6:**
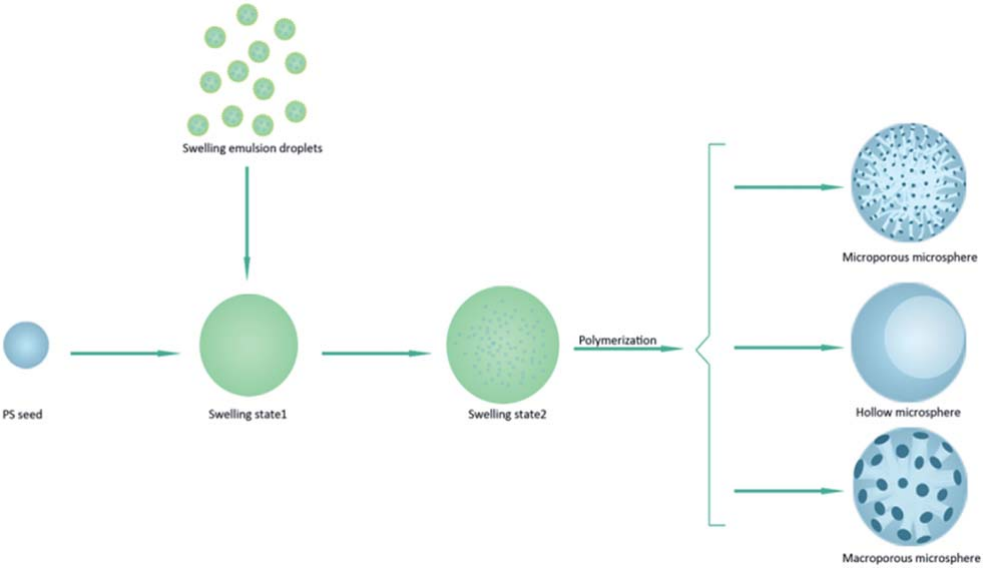
Schematic illustration of preparing microporous, hollow, and macroporous PGMA-DVB particles.

As illustrated in Fig. 6, only a few micelles were brought into the swelling PS seeds when the amount of Span 80 was below 0.6 mL. A small amount of Span 80 was not enough to introduce enough H_2_O to the swelling PS seeds. And the water and Span 80 were nonsolvent for polymer chain. As the addition was increased from 0 to 0.6 mL, the swelling particles absorbed more and more Span 80 and water which made the phase separation between polymer chain and porogen occur earlier, and the obtained particles possess a significantly lower surface area but larger pore size as presented in Table 1.^54^ This was also further confirmed that the absorption of Span 80 increased as the addition of Span 80. As the amount of Span 80 constantly increased, there were more and more H_2_O droplets proceeded to the swelling PS seeds. When the addition of Span 80 reached to 0.9 mL, the swelling particles absorbed many micelles. The micelles presented in the swelling particles were not stable as temperature increased. The surfactant would separate from emulsion, the droplets more easily coalesce and collide.^55^ The polymerization temperature was at 70 °C. In the polymerization process, the internal micelles of the swelling particles took place of random collision and coalescence. And after introduction of Span 80, the swelling particles could also absorbed water from the aqueous phase. This may be the reason that the internal shape of obtained particles was irregular. After polymerization, the large water droplets occupied a large volume. And due to oil phase fabricate the thin polymer shell and internal bulk. The strength of thin polymer shell was too low to break. This was the reason of the Fig. 3a appeared. When addition amount comed to 1.2 mL, more water droplets were introduced into the swelling particles. During the polymerization at 70 °C, the droplets clustered together and the absorption of H_2_O from the aqueous phase shape into one big H_2_O droplets. After completion of the reaction, the obtained particles were hollow structure.

#### 3.3.2 Formation of microspheres with macroporous structure

**Fig. 7:**
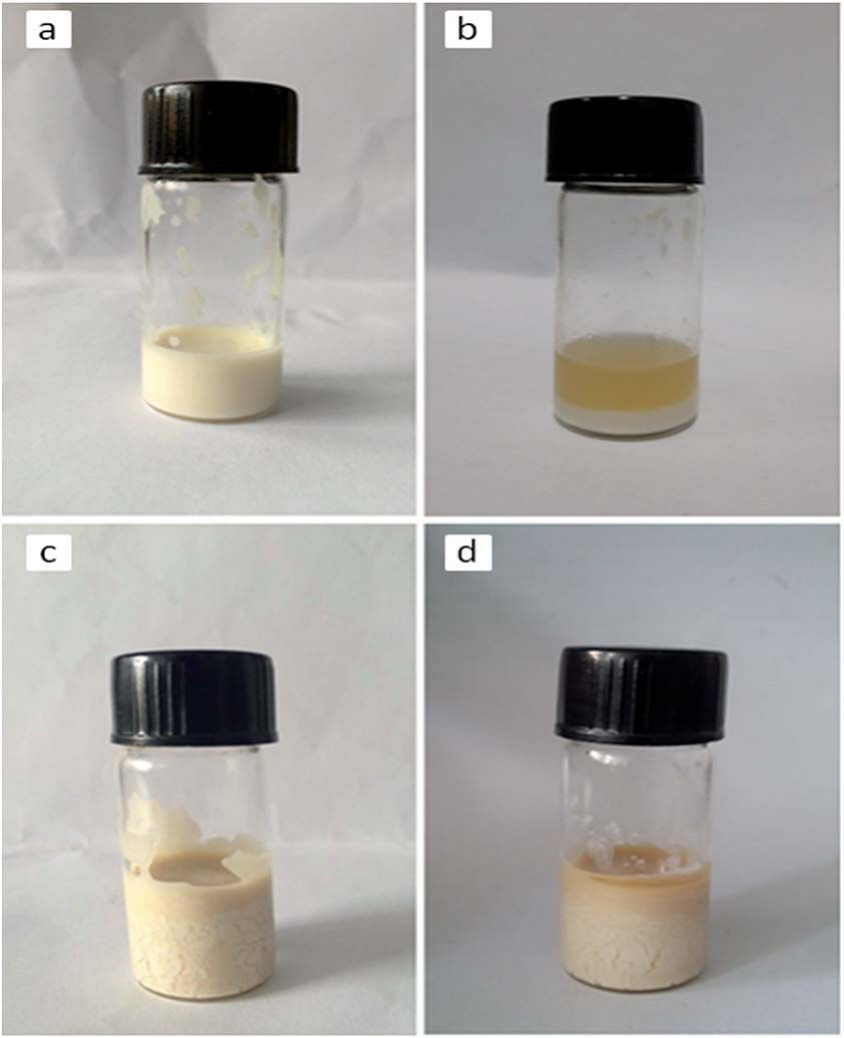
Comparison of emulsion stability: (a) emulsion containing 2 mL toluene, 0.6 mL water and 0.6 mL Span 80; (b) emulsion (a) heated for 10 h at 70 °C; (c) emulsion containing 2 mL toluene, 1.8 mL water and 1.6 mL Span 80; and (d) emulsion (c) heated for 10 h at 70 °C.

As the amount of Span 80 increased, a lot of monomer phase emulsion droplets were absorbed by the swelling particles. The obtained microsphere turned from hollow structure to macroporous. This was mainly caused by that the internal of the swelling particles was supposed as O/W emulsion system and the stability of O/W emulsion system enhanced as the amount of Span 80 increased. It was hard to determine the amount of each component for the swelling progress was complex, however, it's no doubt that the introduction of H_2_O into the system along with increasing amount of Span 80. In order to prove that the O/W emulsion in the swelling particles was more stable as the amount of Span 80 increased, we designed another experiment: 2.0 mL toluene was utilized as the oil phase, 0.6 mL Span 80 and 0.6 mL water was added to the 2.0 mL toluene. Because the more Span 80 meant more water was introduced into system, 1.6 mL Span 80 and 1.8 mL water were similarly added to the 2.0 mL toluene for comparison. They were heated at 70 °C which was as same as the polymerization temperature for 10 h. The result was shown in Fig. 7. The O/W emulsion occurred separation after heating. The amount of separated toluene in the addition of 0.6 mL Span 80 was more than that of the addition of 1.6 mL. It could be concluded that O/W emulsion in the swelling particles was unstable and easy to occur phase separation and its stability depended on the amount of Span 80.

#### 3.3.3 Formation of microspheres with microporous structure

Therefore, the swelling particle constantly absorbed more water. In other words, the non-solvent accounted for large proportion during the polymerization. The phase separation became earlier. So the obtained particles were macroporous (Fig. 3c). But when the absorption of water went beyond the limit of the swelling particles, the swelling particles grew smaller and possessed small microporous structure (Fig. 4b).

## 4. Conclusions

In this study, we develop a new method to prepare hollow porous microsphere, we firstly introduced microsphere into the seeded emulsion polymerization. We found that the amount of Span 80 dramatically affected the morphology of microspheres. The pore size of microsphere was easily controlled by changing the concentration of Span 80. By adjusting Span 80 concentration, polymer microspheres with microporous, macroporous or hollow structure could be obtained. In addition, compared to the previous work, the obtained hollow microspheres had a better monodispersity and uniformity. The microporous and macroporous microspheres can be used as stationary phase for molecular separation. The hollow particles with breach have potential use in drug delivery. The monomer GMA with epoxy groups provided microspheres a lot of possibilities for surface modification.

## Conflicts of interest

There are no conflicts to declare.

## Supplementary Material
